# Targeting senescence in Amyotrophic Lateral Sclerosis: senolytic treatment improves neuromuscular function and preserves cortical excitability in a TDP-43^Q331K^ mouse model

**DOI:** 10.21203/rs.3.rs-6081213/v1

**Published:** 2025-03-26

**Authors:** Jose A. Viteri, Nathan R. Kerr, Charles D. Brennan, Grace R. Kick, Meifang Wang, Arsh Ketabforoush, Harper K. Snyder, Peter J. Moore, Fereshteh B. Darvishi, Anna Roshani Dashtmian, Sindhuja N. Ayyagari, Kelly Rich, Yi Zhu, W. David Arnold

**Affiliations:** Department of Physical Medicine and Rehabilitation, University of Missouri-Columbia, Columbia, MO USA; NextGen Precision Health, University of Missouri-Columbia, Columbia, MO USA; Department of Physical Medicine and Rehabilitation, University of Missouri-Columbia, Columbia, MO USA; NextGen Precision Health, University of Missouri-Columbia, Columbia, MO USA; Department of Physical Medicine and Rehabilitation, University of Missouri-Columbia, Columbia, MO USA; NextGen Precision Health, University of Missouri-Columbia, Columbia, MO USA; Department of Ophthalmology, University of Missouri-Columbia, Columbia, MO USA; Department of Physical Medicine and Rehabilitation, University of Missouri-Columbia, Columbia, MO USA; NextGen Precision Health, University of Missouri-Columbia, Columbia, MO USA; Department of Physical Medicine and Rehabilitation, University of Missouri-Columbia, Columbia, MO USA; NextGen Precision Health, University of Missouri-Columbia, Columbia, MO USA; Department of Physical Medicine and Rehabilitation, University of Missouri-Columbia, Columbia, MO USA; NextGen Precision Health, University of Missouri-Columbia, Columbia, MO USA; Department of Physical Medicine and Rehabilitation, University of Missouri-Columbia, Columbia, MO USA; NextGen Precision Health, University of Missouri-Columbia, Columbia, MO USA; Department of Physical Medicine and Rehabilitation, University of Missouri-Columbia, Columbia, MO USA; NextGen Precision Health, University of Missouri-Columbia, Columbia, MO USA; Department of Physical Medicine and Rehabilitation, University of Missouri-Columbia, Columbia, MO USA; NextGen Precision Health, University of Missouri-Columbia, Columbia, MO USA; Department of Physical Medicine and Rehabilitation, University of Missouri-Columbia, Columbia, MO USA; NextGen Precision Health, University of Missouri-Columbia, Columbia, MO USA; Department of Genetics, Harvard Medical School, Boston, MA USA; Department of Physiology and Biomedical Engineering, Mayo Clinic, Rochester, MN, USA; Robert & Arlene Kogod Center on Aging, Mayo Clinic, Rochester, MN, USA; Department of Physical Medicine and Rehabilitation, University of Missouri-Columbia, Columbia, MO USA; NextGen Precision Health, University of Missouri-Columbia, Columbia, MO USA

**Keywords:** ALS, aging, senescence, senolytics, motor cortex, neuromuscular

## Abstract

Amyotrophic lateral sclerosis (ALS) is a fatal neurodegenerative disorder marked by progressive motor neuron degeneration in the primary motor cortex (PMC) and spinal cord. Aging is a key factor in ALS onset and progression, with evidence suggesting that biological aging—a process involving cellular decline— far outpaces chronological aging in ALS. This promotes senescent cell accumulation—marked by irreversible cell-cycle arrest, impaired apoptosis, and chronic inflammation—disrupting tissue homeostasis and impairing neuronal support functions. Thus, targeting senescence presents a novel therapeutic strategy for ALS.

Here, we investigated the senolytic combination Dasatinib and Quercetin (D&Q) in TDP-43^Q331K^ ALS mice. D&Q improved neuromuscular function and reduced plasma neurofilament light chain, a biomarker of axonal damage. The most pronounced improvement was the improved cortical excitability, accompanied by reductions in senescence and TDP-43 in the PMC. These findings highlight the potential of senolytics to mitigate ALS-related dysfunction, supporting their viability as a therapeutic strategy.

## 1. INTRODUCTION

Amyotrophic lateral sclerosis (ALS) is a fatal age-related neurodegenerative disorder characterized by the progressive degeneration of motor neurons in the primary motor cortex (PMC) and spinal cord [1]. In both genetic and sporadic forms, ALS symptoms often begin with focal muscle weakness that spreads regionally, ultimately leading to generalized muscle atrophy, paralysis, and death, with a median survival of three years after disease onset [1].

The onset of ALS typically occurs in the sixth decade of life [2], emphasizing that aging is a key factor in disease development and progression. In healthy individuals, biological aging—the gradual decline in cellular and physiological function over time—generally progresses in parallel with chronological aging. This process involves molecular changes such as DNA damage, epigenetic modifications, mitochondrial dysfunction, and chronic inflammation [2–4]. However, in ALS, biological aging appears to outpace chronological aging [5], accelerating the accumulation of cellular senescence, marked by irreversible cell-cycle arrest, impaired apoptosis, and persistent cellular inflammation [6–8]. Notably, senescent cells disrupt tissue homeostasis and may drive the spread of neurodegeneration in ALS [9, 10]. In particular, glial support cells like microglia and astrocytes can become senescent, impairing their ability to regulate inflammation and sustain normal motor neuron function [2, 7, 9–13]. Accordingly, senescence has been suggested as a contributing factor in ALS pathology [2, 7, 11].

Given this link between senescence and ALS, reduction of cellular senescence may offer a novel therapeutic approach [7]. To target senescence, senolytics, a class of drugs that selectively eliminate senescent cells, have shown promise in alleviating aging-related pathologies [7, 14–16]. Among the most studied is the combination of Dasatinib and Quercetin (D&Q), which synergistically target and suppress anti-apoptotic pathways and senescence-driven inflammation [16–19]. D&Q has demonstrated efficacy in preclinical models of Alzheimer’s disease [20], traumatic brain injury [21], and age-related metabolic dysfunction [22]. Despite their potential, senolytics remain entirely unexplored in ALS [7], presenting an opportunity to assess their impact on disease onset and progression.

To investigate the therapeutic potential of senolytics, we tested the effects of D&Q in TDP-43^Q331K^ mice, a model that replicates key ALS features, including motor neuron loss, glial dysfunction, and motor deficits [23, 24]. This model mirrors the TDP-43-driven pathology found in most sporadic ALS cases [23, 24], making it relevant for translational research. We longitudinally treated TDP-43^Q331K^ mice with D&Q over 15 weeks, starting at ~ 2.3 months of age, and assessed its effects on motor strength, coordination, neuromuscular function, and cortico-muscular excitability. Additionally, we performed longitudinal evaluations of neuronal health markers and terminal analyses of senescence-associated molecular and cellular changes.

Together these studies provide evidence that D&Q has therapeutic potential in ALS by reducing senescence, preserving neuromuscular function, and restoring cortical excitability.

## 2. METHODS

### 2.1 Animals

**Figure 1:**
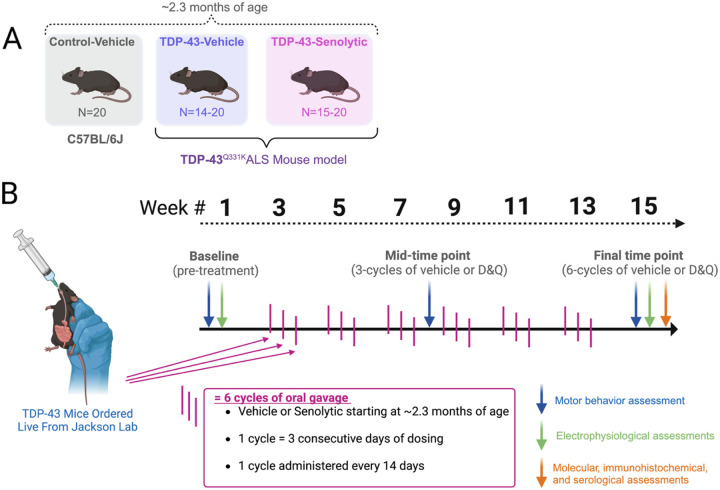
Experimental design and timeline for evaluating the effects of senolytic treatment in TDP-43 ALS mice. **(A)** Experimental groups included control-vehicle mice (C57BL/6J, N = 20), TDP-43^Q331K^ vehicle-treated mice (N = 14–20), and TDP-43^Q331K^ senolytic-treated mice (N = 15–20). All mice were approximately ~ 2.3 months old at the start of the study. Senolytic-treated groups received a combination of Dasatinib and Quercetin (D&Q), while vehicle groups received the equivalent vehicle solution. **(B)** Experimental timeline showing three main time points of assessment. D&Q or vehicle treatment began at approximately ~ 2.3 months of age (after baseline measures were performed), administered via oral gavage in 6 cycles, with each cycle consisting of 3 consecutive days of dosing every 14 days. Assessments included motor behavior (blue arrows), in-vivo electrophysiology (green arrows), and molecular, immunohistochemical, and serological assessments (orange arrows), conducted at different combinations at baseline (pre-treatment; ~2.3 months of age), a mid-time point (3 cycles of vehicle or senolytics; ~4.5 months of age), and at a final time point (6 cycles of vehicle or senolytics; ~6 months of age). The experimental design highlights longitudinal dosing and assessments to evaluate senolytic efficacy.

All animal experiments were approved by the Institutional Animal Care and Use Committee (IACUC) at the University of Missouri – Columbia (protocol #39905). Control C57BL/6J (Jackson Laboratories) and TDP-43^Q331K^ (C57BL/6J background; Jackson Laboratories) mice were used for all experiments. At the beginning of experiments, mice ranged in age from 1.2 to 3.2 months, with an average age of ~ 2.3 months (Fig. 1A). Variation in age was due to the availability of TDP-43^Q331K^ mice from Jackson laboratories. TDP-43^Q331K^ were randomized to two groups: TDP-43-vehicle (TDP-43^Q331K^, N = 14–20), and TDP-43-senolytics (TDP-43^Q331K^, N = 15–20). We also included a vehicle-control (C57BL/6J, N = 14–20). All groups were initially balanced for sex (50% female) at baseline (Fig. 1 A). Sample sizes for the TDP-43 groups changed over the experimental timeline due to mortality following anesthesia ketamine (100 mg/kg) and xylazine (7.5 mg/kg) administration during baseline electrophysiological experiments (pre-treatment) (Fig. 1B). At the mid-time point (3 cycles of vehicle or senolytics), the TDP-43-vehicle group was reduced from N = 20 to N = 14 and this sample size continued into the final time point (male N = 6, Female N = 8). At the mid-time point, the TDP-43-senolytics group was reduced from N=20 to N = 15 and this sample size continued into the final time point (male N = 7, Female N = 8).

### 2.2 Genotyping of animals

TDP-43^Q331K^ were genotyped with the human huTDP-43 gene (forward 5’-AGAGGTGTCCGGCTGGTAG-3’, reverse 5’-CCTGCACCATAAGAACTTCTCC-3’) with a polymerase chain reaction (PCR) and utilizing standard procedures [23].

### 2.3 Senolytic treatment

Figure 1B summarizes the experimental timeline and cadence of treatment. Mice in the senolytic group received Dasatinib (5 mg/kg; Sigma-Aldrich, St. Louis, MO, USA) and Quercetin (50 mg/kg; Thermo Fisher, Waltham, MA, USA) in a solution of 10% ethanol, 30% polyethylene glycol 400, and 60% Phosal 50 PG (Sigma-Aldrich). Vehicle-treated mice received an equal volume of the vehicle solution. Dosing began after baseline assessments of behavior and in-vivo electrophysiology (Fig. 1B; methods 2.4–2.7) and occurred every 14 days in three-day cycles for a total of six cycles, starting at approximately 2.3 months of age. Individuals administering senolytics or vehicle treatment doses were unblinded to genotype and group but were not involved in data collection.

### 2.4 Assessment of all-limb grip strength

For this and all subsequent described outcome measures, experimenters were blinded to group. All-limb grip strength was assessed using a grip force transducer (Bioseb Model GT3, Pinellas Park, FL, USA) [25] (Fig. 1B). Mice were gently grasped by the tail and placed on a metal grid, ensuring all four limbs made contact. A steady horizontal tug on the tail was applied, and the force exerted in grams was recorded. Each mouse underwent three trials, and the average grip strength was normalized to body weight (grams/grams of body weight) and recorded as the final value. Baseline behavioral assessments, including grip strength and coordination (methods 2.5), were conducted at an average age of 2.3 months and repeated every 45 days at a mid-time point and at a final time point (Fig. 1B).

### 2.5 Assessment of motor coordination.

Motor coordination was assessed using the rotarod test (BX-ROD, Bioseb, Pinellas Park, FL, USA) as described in other studies using the same ALS model [23]. Mice were placed on a rotating rod and allowed to acclimate for 30 seconds while the rod rotated at 4 rpm. The test protocol consisted of a ramped increase in speed from 4 to 40 rpm over 300 seconds [23]. The latency to fall was recorded as the time taken for the mouse to fall from the rod, and the test was repeated for a second trial. The highest latency time from the two trials was normalized to body weight (time/gram) and recorded as the final value.

### 2.6 In-vivo electrophysiology to assess neuromuscular excitability.

Compound muscle action potential (CMAP) amplitude, average single motor unit potential (SMUP) amplitude, repetitive nerve stimulation (RNS) decrement, and motor unit number estimation (MUNE) of the gastrocnemius were measured following protocols as previously described [26, 27]. Figure 4 shows a representative figure depicting setup for these recording. Mice were deeply sedated with an intraperitoneal injection of ketamine (100 mg/kg) and xylazine (7.5 mg/kg), placed on a 37°C heating pad, and positioned prone with the right hindlimb secured with medical tape at a 90° relative to the tail (Fig. 3E). Eye gel was applied to prevent dryness. Two ring electrodes (MFI Medical, San Diego, CA, USA) with conductive gel were positioned around the gastrocnemius (Electrode 1) and calcaneus (Electrode 2). Stimulating needle electrodes (anode and cathode) were placed subcutaneously near the sciatic nerve in the thigh, and a ground electrode was taped to the tail. Electrophysiological recordings were performed using a Sierra Summit system (Cadwell, Kennewick, WA, USA).

CMAP amplitudes, representing a summated recording of muscle excitation and an indicator of neuromuscular excitability, were recorded by applying a supramaximal stimulus to the sciatic nerve. CMAP amplitudes were quantified using peak-to-peak amplitude. Recording sensitivity was set at 10 mV/division, with a time sensitivity of 1 ms/division. SMUPs were recorded as described by Arnold et., 2015, Kerr et al., 2025., 2019 [26, 27], to assess average single motor unit size. A gradually increasing stimulus was applied at 1 Hz to elicit 10 incremental all-or-none responses. SMUP values were calculated as the peak-to-peak amplitude of the 10th SMUP divided by the number of responses (10). Sensitivity was set at 200 μV/division and 1 ms / division.

MUNE was estimated using CMAP and SMUP values as previously described (MUNE = CMAP/SMUP) [26]. RNS decrement, an assessment of NMJ transmission, was measured by recording repetitive CMAP responses during a train of 10 repetitive stimulations at 50 Hz. The percentage decrement was calculated as [(Amplitude of 10th response - Amplitude of 1st response)/Amplitude of 1st response] × 100%, using the responses at 50 Hz.

Baseline electrophysiological assessments (pre-treatment) were conducted at an average age of 2.3 months and repeated at the final time point (6 cycles of vehicle or senolytics) (Fig. 1B). Following treatment randomization, assessments requiring anesthesia were limited to the final time point to minimize mouse loss due to mortality in 11 male TDP-43^Q331K^ mice during recovery from ketamine at baseline (see methods 2.1).

### 2.7 In-vivo electrophysiology to assess cortico-muscular connectivity

Motor-evoked potentials (MEPs) were recorded following a protocol similar to Kerr et al., 2025 [26]. Like CMAPs, MEP amplitudes reflect the totality of action potentials elicited from the muscle, but are a function of cranial or cervical stimulation, providing insights into the excitability and connectivity of the muscle to the PMC or corticospinal tracts. The setup for MEP recording (Fig. 4) was similar to that of CMAPs, with the primary difference being the placement of stimulating electrodes.

For cranial MEPs, stimulating electrodes (MFI Medical, San Diego, CA, USA) were placed subcutaneously on opposite sides of the cranium, 3–4 mm left and right of the bregma (Fig. 4), to generate an electric field around the PMC and motor area [28]. For cervical MEPs, electrodes were placed subcutaneously on opposite sides of the vertebral column near the C2–C3 vertebrae (Fig. 3E), generating an electric field around the corticospinal tracts [26, 29]. A supramaximal stimulus was applied to evoke maximal MEPs from the gastrocnemius muscle, with amplitudes recorded as the peak-to-peak values that were both maximal and stable. Cranial and cervical MEPs were confirmed by latency, with cranial MEPs averaging 15–17 ms and cervical MEPs 5–10 ms [26]. Amplitude recording sensitivity was set at 500 μV/division, and time sensitivity at 5 ms/division.

Final MEP values were normalized as the MEP amplitude (mV) divided by the CMAP amplitude (mV) [8]. This normalization was performed to remove contribution of neuromuscular excitability loss, isolating the excitability of the PMC or corticospinal tracts. As described in methods 2.6, assessments were limited to two time points (Baseline and Final).

### 2.8 In-vivo electrophysiology to assess contractile torque

Plantar flexion contractility torque was measured following previously published protocols [30, 31]. This method assessed torque production as a readout of neuromuscular and muscle contractile function following peripheral nerve stimulation. Figure 4 shows a representative figure depicting setup for these recordings. After completing CMAP and MEP measurements, the right hindlimb foot was affixed to a force transducing pedal using medical tape, with the tibia aligned at a 90° angle. The femoral condyles were secured with a clamp to ensure rotation occurred only at the ankle. Subcutaneous needle electrodes (MFI Medical, San Diego, CA, USA) were placed around the tibial branch of the sciatic nerve. Force recordings were conducted using a 1305A whole animal system (Aurora Scientific Inc, Canada). Twitch contractility torque was measured using a single 0.20 ms supramaximal stimulus, while tetanic torque was measured with a 1 s stimulus train at 125 Hz. Final contractility values were recorded in mN-m and normalized to body weight. As described in methods 2.6, assessments were limited to two time points (Baseline and Final).

### 2.9 Digital PCR and biochemical analyses

We quantified RNA expression of the senescent markers, *P21, BCL1, IL1B, BCL2, P53, P16, BCLxl, and BCLw,* as well as the human TDP-43 transcript in the mouse cortex of a subset of mice (N = 7 per group, approximately 50% female). At the completion of the final time point (Fig. 1B), mice were cervically dislocated while deeply anesthetized, the brain harvested, the cortex removed, wrapped in aluminum foil, flash frozen in liquid nitrogen, and then stored at −80°C. Total RNA was isolated first by mixing cortex tissue with of 400 μL Trizol (Thermo Fisher Scientific, Waltham, Massachusetts, USA), 80 μL chloroform, homogenizing beads, and then centrifuged and homogenized at 12000 × g. The separated clear aqueous phase was then carefully removed and pipetted into a column extraction kit per the manufacturer’s instructions (Quick-RNA MicroPrep Kit, Zymo Research). From the final elution volume, we took the necessary amount to achieve a 1 μg total amount of RNA. We then used this volume to synthesize cDNA from tissue using the SuperScript IV VILO RT in a 20 μL reaction per the manufacturer’s instructions (Thermo Fisher Scientific, Waltham, Massachusetts, USA). This final volume was then resuspended to 310 μL with water to achieve an 8 μg / μl concentration of RNA. We used this final volume as the cDNA template for dPCR reactions. cDNA was stored at −20°C

For dPCR reactions, we used pre-designed and pre-validated primer/probe pairs purchased from Thermo Fisher Scientific (Waltham, Massachusetts, USA). Assay IDs for each primer/probe pair were: Mm00432359_m1 *(BCL1),* Mm04205640_g1 *(P21),* Mm01731290_g1 (P53), Mm00477631_m1 *(BCL2)*. Mm00434228_m1 *(IL1B),* Mm00437783_m1 *(BCLXL),* Mm00432054_m1 *(BCLW),* and Hs00606522_m1 *(hTARDBP)*. To multiplex assays in each dPCR reaction, assays were custom-validated by the manufacturer (Thermo Fisher Scientific, Waltham, Massachusetts, USA) to prevent interaction between fluorescent probes. dPCR reactions were performed in a QuantStudio Absolute Q Digital PCR System (Thermo Fisher Scientific, Waltham, Massachusetts, USA). dPCR reactions consisted of a 1X Applied Biosystems Absolute Q DNA Digital PCR Master Mix (2 μL) (Thermo Fisher Scientific, Waltham, Massachusetts, USA), 0.5X of each primer/probe mix (0.5 μL), 1 μL of cDNA sample, and nuclease free water for a total reaction volume of 10 μL. The assays were conducted on a MAP16 plate (Thermo Fisher Scientific, Waltham, Massachusetts, USA) and we ran the assays with the following thermal cycling protocol: preheating step of 96°C for 10 min, 40 cycles of denaturing and annealing steps of 96°C for 5 sec, and 60°C for 15 sec respectively. After reactions were completed, analysis for final dPCR copies was done on QuantStudio Absolute Q Software version 6.3 (Thermo Fisher Scientific, Waltham, Massachusetts, USA). Copy # / μL for each assessed transcript was normalized to (divided by) the copy # / μL of the mouse housekeeping gene GAPDH (Mm99999915_g1) for each individual sample.

Blood serum was collected at timepoints 1–3 to analyze neurofilament light (NfL), a biomarker of axonal damage. Using a hypodermic needle, blood was drawn from the saphenous vein, with a maximum of 100 μL collected per sample. To prevent coagulation, 1 μL of heparin was added per 100 μL of blood. Samples were centrifuged at 2000 × g for 20 minutes to separate the plasma, which was then flash frozen in liquid nitrogen and stored at −80°C. Plasma samples were shipped overnight to RayBiotech (Peachtree Corners, GA, USA) for SIMOA analysis per the manufacturers protocol, with NfL levels reported in pg / mL.

### 2.10 Immunohistochemistry

Immunohistochemical assessments were performed similar to Kerr et al., 2023 [32][32]. At the final time point, a subset of mice from each group (N = 4, 50% female) underwent cardiac perfusion with normal saline. Brains were then harvested, fixed in 4% paraformaldehyde (PFA) for 24 hours, and transferred to 30% sucrose in 1X PBS until equilibrated. Brains were then frozen on dry ice, embedded in OCT, and sectioned at 30 μm using a cryostat microtome to obtain coronal sections spanning the PMC (+ 1.0 mm to −1.5 mm from the bregma). Sections were stored in PBS at 4°C.

For immunohistochemistry (IHC), every 6th section was selected for a total of six sections per animal, with one additional section per animal designated as a negative control (no primary antibody, but secondary antibody added). Sections were washed three times in PBS (10 minutes each), blocked for 1 hour in 10% Normal Donkey Serum (NDS) with 0.3% Triton-PBS, and washed again three times in PBS. Sections were incubated overnight at 4°C with primary antibodies diluted in 1% NDS and 0.3% Triton-PBS. Primary antibodies: rabbit anti-CTIP2 (1:700; Abcam, ab240636), mouse anti-hTDP-43 (1:2000; Thermo Fisher Scientific, MA5–47420), goat anti-P53 (1:3000; R&D Systems, AF1355), rabbit anti-P21 (1:3000; Proteintech, 10355–1-AP), mouse anti-BCL2 (1:3000; Proteintech, 68103–1-Ig), goat anti-IBA1 (1:1000; abcam, ab289874).After incubation, sections were washed three times in PBS (10 minutes each) and then incubated for 1.5 hours in the dark with secondary antibodies diluted 1:500 in 1% NDS and 0.3% Triton-PBS. Secondary antibodies included Donkey-anti-Rabbit IgG 647 (A-31573), Donkey-anti-Mouse IgG 488 (A-21202), Donkey-anti-Goat IgG 405 (A48259), and Donkey-anti-Goat IgG 546 (A-11056) (all from Thermo Fisher Scientific). Sections were washed again (three 10-minute washes in PBS in the dark), mounted onto gelatin-coated slides, covered with ProLong Gold Antifade (Thermo Fisher Scientific, P36930), and sealed with coverslips and nail polish. Slides were stored at 4°C in the dark.

Images were acquired using an inverted IX83 Olympus widefield microscope equipped with a Hamamatsu camera at 20x magnification and processed with Olympus cellSens software. Imaging was performed in Layer V of the PMC from both hemispheres, and post-hoc analysis was conducted with ImageJ software. For primary antibody cassette 1, counts of CTIP2-positive cells, mean fluorescence intensity (MFI) of TDP-43, and colocalization of CTIP2 and TDP-43-positive cells (overlapping pixel MFI then normalized to CTIP2-positive cells in layer V) were measured. For primary antibody cassette 2, the MFI of P53, P21, and BCL2 was evaluated. For primary antibody cassette 3, we performed two analyses. First, we quantified counts of IBA1 positive cells, as well as assessing morphological microglial changes (area, perimeter, circularity) to assess microglial activation as previously described [33]. We then quantified MFI of colocalized pixels of IBA1 + P21, IBA1 +TDP43, and IBA1 + P21 + TDP43. For each animal, n = 6 sections were placed on each slide for immunohistochemical assessments. Measurements were taken from each hemisphere; thus, each animal yield a total of 10–12 data points for each immunohistochemical assessment. Statistical analyses were adjusted accordingly to account for within group differences (see methods 2.11 below). All measurements were done in layer V of the mouse PMC. Layer V was identified anatomically as described in Viteri et al., 2024 [34].

### 2.11 Statistical analysis

All statistical analyses were performed using GraphPad Prism 10 (GraphPad Version 10.1.1 (270) Software, San Diego, CA, USA). Data that included two factors (animal group and time point) such as weight measurements, electrophysiological assessments, behavioral assessments, and NfL quantification were analyzed using a two-way ANOVA. For dPCR data, a one-way ANOVA was used to assess differences between groups. For immunohistochemical assessments (pixel colocalization, mean fluorescence intensity, cell counts, morphological assessments) we used nested one-way ANOVAs to account for within group differences, given that multiple immunohistochemical measurements were performed from the same animal (N=4 per group, n = ~12 per immunohistochemical assessment; see methods 2.10). Post-hoc analyses for all multiple-comparisons was done using Tukey’s Honestly Significant Difference (HSD) test. Data normality was assessed using the Shapiro-Wilk test. All group data are expressed as means ± standard deviation (SD), and statistical significance was determined using an alpha of p < 0.05.

## 3. RESULTS

### 3.1 The senolytics D&Q improve the motor function and neuromuscular excitability of TDP-43^Q331K^ mice.

**Figure 2:**
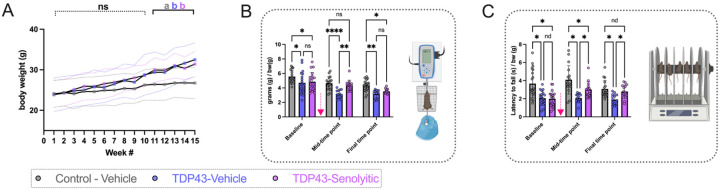
Senolytic treatment improves motor function in TDP-43^Q331K^ ALS mice. **(A)** Longitudinal body weight measurements over 15 weeks of the study. No significant differences were observed between TDP-43-vehicle and TDP-43-senolytic mice at any time point, though both groups exhibited significantly higher body weights compared to control-vehicle mice after week 11. Data are presented as group means ± SD. **(B)** All-limb grip strength normalized to body weight (grams/grams of body weight) was assessed across three time points. At the mid-time point, after three cycles of senolytic treatment, TDP-43-senolytic mice showed significantly improved grip strength compared to TDP-43-vehicle mice, but this improvement was lost by the final time point. Control-vehicle mice consistently exhibited higher grip strength than both TDP-43 groups. Grip strength was measured using a force transducer, as illustrated in the inset diagram. **(C)** Latency to fall during the rotarod test, normalized to body weight (seconds/grams of body weight), was measured to evaluate motor coordination. TDP-43-senolytic mice exhibited improved rotarod performance compared to TDP-43-vehicle mice at the mid-time point and at the final time point, but their performance remained lower than control-vehicle mice. Rotarod setup is illustrated in the inset diagram. Significance: *P < 0.05, **P < 0.01, ****P < 0.0001, ns = not significant. Data are presented as means ± SD. The pink arrow indicates when mice received either vehicle or D&Q.

Mouse body weights were recorded weekly (Fig. 2A). There was no significant interaction between factors (group and time) (P = 0.3094), but both main effects (group and time) significantly influenced weight [F(14, 645) = 14.14, P< 0.0001; F(2, 645) = 27.72, P< 0.0001]. Groups showed no weight differences until week 11. At this point, TDP-43-vehicle and TDP-43-senolytic groups had similar weights (29.4 ±5 g vs. 30±3 g, P = 0.6786), both heavier than controls (26.4 ±4 g; P = 0.0252, P = 0.0067). This continued through week 15, with TDP-43 groups still heavier than controls (32.5 ± 4.2 g and. 31.4 ± 3.1 g vs. 26.8 ±4 g; P< 0.0001, P = 0.0004).

Motor strength and coordination were assessed at three time points (Fig. 2B, C). Grip strength showed no significant interaction between factors (group and time) (P = 0.1780), but both main effects (group and time) were significant [F(2, 146) = 20.98, P < 0.0001; F(2, 146) = 32.15, P< 0.0001]. At baseline (pre-treatment), TDP-43 groups had weaker grip strength than controls (4.7 ±1.5 g/bw and 4.9 ±1.3 g/bw vs. 5.6 ± 0.8 g/bw; P = 0.0104, P = 0.0453). At the mid-time point (three treatment cycles), TDP-43-vehicle mice remained impaired (3.1 ± 0.5 g/bw vs. 4.7 ± 0.9 g/bw, P < 0.0001), while TDP-43-senolytic mice improved (4.4 ± 0.7 g/bw, P = 0.0013) and matched controls (P = 0.6625). At the final time point (six treatment cycles), TDP-43-senolytic mice lost improvements, aligning with TDP-43-vehicle (3.5 ± 0.3 g/bw vs. 3.2 ± 0.6 g/bw, P = 0.5460) and remaining lower than controls (P = 0.0318).

Rotarod performance showed a significant interaction between factors (group and time) (P = 0.0313) and both main effects (group and time) were also significant [F(2, 143) = 3.570, P = 0.0307; F(2, 146) = 31.82, P = 0.0307] (Fig. 2C). At baseline (pre-treatment), TDP-43 groups performed worse than controls (2.0 ± 0.8 rotarod/bw and 2.1 ± 0.8 rotarod/bw vs. 3.7 ±1.5 rotarod/bw; P < 0.0001, P< 0.0001). At the mid-time point (three treatment cycles), TDP-43-vehicle mice remained impaired compared to controls (2.1 ±0.5 rotarod/bw vs. 4.1 ±1.6 rotarod/bw, P < 0.0001), but TDP-43-senolytic mice improved compared to TDP-43-vehicle mice (3.0 ± 0.9 rotarod/bw vs. 2.1 ± 0.6 rotarod/bw, P = 0.0033), though still more impaired than controls (P = 0.0151). At the final time point (six treatment cycles), TDP-43-vehicle mice remained impaired compared to controls (1.9 ± 0.6 rotarod/bw vs. 3.1± 0.9 rotarod/bw, P = 0.0017), while TDP-43-senolytic mice maintained improvements compared to TDP-43-vehicle mice (2.8 ± 0.7 rotarod/bw vs. 1.9± 0.6 rotarod/bw, P = 0.0201) and were no longer different from controls (P = 0.4280).

**Figure 3:**
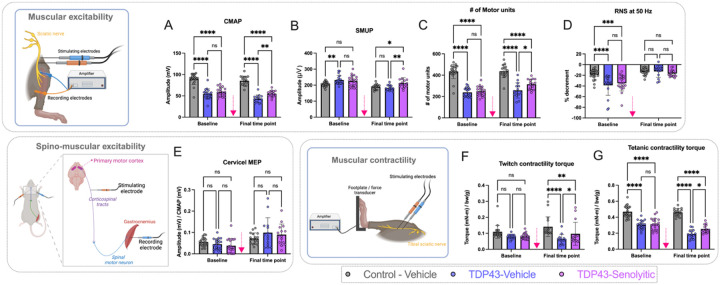
Senolytic treatment improves neuromuscular excitability and contractility in TDP-43^Q331K^ALS mice. **(A)** TDP-43-senolytic mice exhibited significant improvements in compound muscle action potential (CMAP, a measure of summated muscle excitability) amplitudes compared to TDP-43-vehicle mice at the final time point, though values remained lower than control-vehicle mice. **(B)** Single motor unit potential (SMUP, a measure of NMJ collateral sprouting and remodeling) amplitudes were significantly higher in TDP-43-senolytic mice compared to TDP-43-vehicle mice at the final time point. **(C)** TDP-43-senolytic mice showed increased motor unit numbers (MUNE, estimated from CMAP and MUNE values) compared to TDP-43-vehicle mice at the final time point but remained lower than control-vehicle mice. **(D)** While both TDP-43 groups exhibited RNS (Repetitive nerve stimulation decrement at 50 Hz, a measure that quantified NMJ transmission) decrements at baseline, TDP-43-senolytic mice showed no differences in RNS decrement compared to TDP-43-vehicle or control-vehicle groups at the final time point. **(E)** TDP-43-senolytic mice exhibited no significant improvements in cervical motor-evoked potentials (MEPs, a measure of corticospinal-muscular connectivity) compared to other groups at both time points. **(F)** TDP-43-senolytic mice exhibited significant improvements in twitch contractility (a measure of instantaneous force production) compared to TDP-43-vehicle mice at the final time point, though values remained lower than controls. **(G)** TDP-43-senolytic mice displayed enhanced tetanic contractility (a measure of sustained force production) compared to TDP-43-vehicle mice at the final time point, but performance did not reach control-vehicle levels. Illustrations depict the experimental setup for neuromuscular excitability (**top-left**), cervical MEPs (**bottom-left**), and neuromuscular contractility (**bottom-middle**) assessments, showing electrode placements and recording methods. Significance: *P < 0.05, **P < 0.01, ****P < 0.0001, ns = not significant. Data are presented as means ± SD. A pink arrow indicates when mice received either vehicle or D&Q.

Neuromuscular connectivity and excitability were assessed via CMAP, SMUP, MUNE, RNS % decrement, and cervical MEP amplitudes (Fig. 3). CMAP, a measure of summated muscle excitability following peripheral nerve stimulation (Fig. 3A), showed no significant interaction between factors (group and time) (P = 0.2407) but significant main effects (group and time) [F(1, 100) = 8.886, P = 0.0036; F(2, 100) = 127.3, P< 0.0001]. At baseline (pre-treatment), CMAP amplitudes were similar between TDP-43 groups (54.5 ± 12.1 mV and 58.5 ± 10.4 mV, P = 0.4828) but lower in TDP-43 than controls (89.6 ± 12.9 mV, P < 0.0001). By the final time point (six treatment cycles), TDP-43-vehicle remained impaired compared to controls (43.0 ± 9.2 mV vs. 85.4 ± 10.9 mV, P < 0.0001), while TDP-43-senolytic improved compared to TDP-43-vehicle mice (55.18 ± 7.4 mV vs. 43.0 ± 9.2 mV, P = 0.0085) but remained lower than controls (P <0.0001).

SMUP, a measure of average motor unit size (Fig. 3B), showed a significant interaction between factors (group and time) (P = 0.0052) and main effects (group and time) [F(1, 100) = 28.58, P < 0.0001; F(2, 100) = 6.529, P = 0.0022]. At baseline (pre-treatment), SMUP amplitudes were similar across TDP-43 groups (232.2 ± 27.8 μV and 225.5 ± 34.5 μV, P = 0.6683), with TDP-43-vehicle higher than controls (P = 0.0055), while TDP-43-senolytic showed no difference with controls (P = 0.0509). By the final time point (six treatment cycles), SMUP was higher in TDP-43-senolytic than in TDP-43-vehicle (213.6 ± 32.8 jV vs. 182.0 ± 18.0 jV, P = 0.0032) and controls (189.2 ± 16.4 jV, P = 0.0210), while TDP-43-vehicle was unchanged from controls (P = 0.7094).

MUNE, an estimate of the number of functional motor units innervating a muscle that is calculated from CMAP and SMUP (Fig. 3C), showed no interaction between factors (group and time) (P = 0.0526) but significant main effects (group and time) [F(1, 100) = 6.777, P = 0.0106; F(2, 100) = 113.2, P< 0.0001]. At baseline (pre-treatment), MUNE was similar in TDP-43 groups (238.9 ± 42.5 and 249.2 ± 44.8, P = 0.8365) but lower than controls (434.9 ± 66.6, P < 0.0001). By the final time point (six treatment cycles), MUNE remained reduced in TDP-43 groups compared to controls (257.6 ± 71.8 vs. 436.8 ± 59.6, P < 0.0001; TDP-43-senolytic: 314.7 ±48.2, P< 0.0001), though TDP-43-senolytic improved over TDP-43-vehicle (P = 0.0202).

RNS % decrement, a measure of the reliability of NMJ transmission (Fig. 3D), showed a significant interaction between factors (group and time) (P = 0.0021) and main effects (group and time) [F(1, 100) = 46.31, P< 0.0001; F(2, 100) = 7.312, P = 0.0011]. At baseline (pre-treatment), TDP-43 groups had similar decrements (−38.8% ± 18.7 and - 34.9% ± 14.5, P = 0.5795), both greater than controls (−18.8% ± 9.7, P< 0.0001 and P = 0.0002). By the final time point (six treatment cycles), TDP-43 groups remained comparable (−12.8% ± 11.2 and - 16.8% ± 6.6, P = 0.6585), with no differences from controls (−13.7%, P = 0.9758 and P = 0.7634).

Cervical MEP amplitudes, measuring corticospinal-muscular excitability (Fig. 3E), showed no interaction between factors (group and time) (P = 0.0843), and only time had a significant main effect [F(1, 100) = 28.98, P < 0.0001; F(2, 100) = 0.5640, P = 0.5707]. At baseline (pre-treatment), TDP-43 groups had similar amplitudes (0.05 ± 0.03 and 0.04 ± 0.03 mV/CMAP, P = 0.8052) and did not differ from controls (0.05 ± 0.02 mV/CMAP; P = 0.7453, P = 0.3325). By the final time point (six treatment cycles), neither TDP-43- vehicle (0.1 ± 0.07 vs. 0.07 ± 0.03 mV/CMAP, P = 0.1330) nor TDP-43-senolytic (0.1 ± 0.05 mV/CMAP, P = 0.3777) differed from controls, with no differences between TDP-43 groups (P = 0.8159).

Plantar flexion force after tibial sciatic nerve stimulation was measured. Twitch contractility torque (Fig. 3F) showed no interaction between factors (group and time) (P = 0.0735) but significant main effects (group and time) [F(1, 100) = 1.670, P< 0.0001; F(2, 100) = 14.48, P < 0.0001]. At baseline (pre-treatment), TDP-43-vehicle and TDP-43-senolytic mice had similar twitch contractility (0.08 ± 0.01 and 0.08 ± 0.02 mN-m/BW, P = 0.9110), both comparable to controls (0.11 ± 0.04 mN-m/BW, P = 0.0810). By the final time point (six treatment cycles), TDP-43-vehicle mice had lower twitch contractility than controls (0.06 ± 0.03 vs. 0.14± 0.06 mN-m/BW, P < 0.0001), as did TDP-43-senolytic mice (0.09 ± 0.07 mN-m/BW, P = 0.0099), though they improved over TDP-43-vehicle (P = 0.0371).Tetanic contractility torque (Fig. 3G) showed a significant interaction between factors (group and time) (P = 0.0016) and main effects (group and time) [F(1, 100) = 25.88, P < 0.0001; F(2, 100) = 131.8, P < 0.0001]. At baseline (pre-treatment), TDP-43 groups had similar tetanic contractility (0.31 ± 0.05 and 0.31 ± 0.07 mN-m/BW, P = 0.9453), both lower than controls (0.47 ± 0.07 mN-m/BW, P < 0.0001). By the final time point (six treatment cycles), TDP-43-vehicle mice remained impaired compared controls (0.2 ± 0.05 vs. 0.5± 0.05 mN-m/BW, P < 0.0001), as did TDP-43-senolytic mice (0.3 ± 0.05 mN-m/BW, P < 0.0001), though they improved over TDP-43-vehicle (P = 0.0173).

### 3.2 The senolytics D&Q improve cortico-muscular excitability TDP-43^Q331K^ mice.

**Figure 4:**
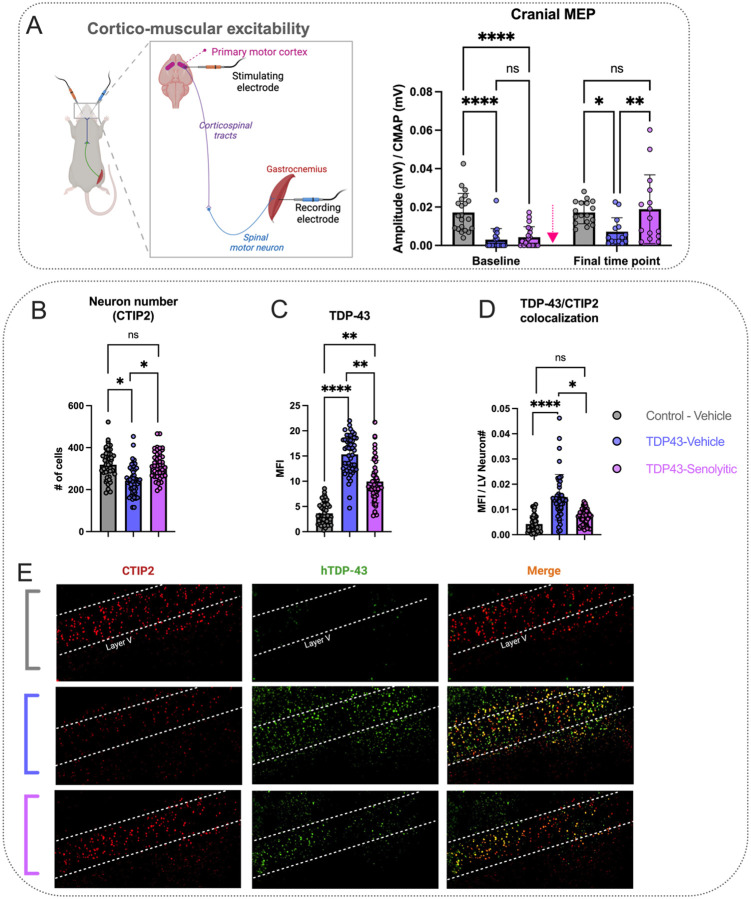
Senolytic treatment enhances cortico-muscular excitability and neuron number preservation in TDP-43^Q331K^ ALS mice. **(A)** Cranial motor-evoked potentials (MEPs), reflecting cortico-muscular excitability and connectivity, were significantly improved in TDP-43-senolytic mice compared to TDP-43-vehicle mice at the final time point and were no longer significantly different from those in control-vehicle mice. The illustration depicts the experimental setup for cranial MEPs, showing stimulation of the PMC and recording of muscle responses from the gastrocnemius. **(B)** CTIP2-positive neurons (red), a marker for layer neurons, are reduced in TDP-43-vehicle mice compared to controls. Senolytic treatment significantly increases CTIP2-positive neuronal density compared to TDP-43-vehicle mice, and levels are not significantly different from controls. **(C)** Human-specific TDP-43 (hTDP-43) expression is significantly elevated in TDP-43-vehicle mice compared to controls. Senolytic treatment reduces hTDP-43 mean fluorescence intensity (MFI) relative to TDP-43-vehicle mice but does not restore levels to those of controls. **(D)** Colocalization analysis of CTIP2 and hTDP-43 shows significantly increased overlap in TDP-43-vehicle mice, indicating elevated TDP-43 pathology in layer V neurons. Senolytic treatment significantly reduces TDP-43/CTIP2 colocalization compared to TDP-43-vehicle mice, though it remains elevated relative to controls. (E) Representative images show CTIP2, hTDP-43, and merged staining in layer V. Significance: *P < 0.05, **P < 0.01, ****P < 0.0001, ns = not significant. Data are presented as means ± SD. A pink arrow indicates when mice received either vehicle or D&Q. If no pink arrow is present, measurements were done terminally at the final time point (Panels B-D).

Cranial MEP amplitudes, a measure of cortical excitability (Fig. 4A), showed a significant interaction between factors (group and time) (P = 0.0041) and main effects (group and time) [F(1, 100) = 12.36, P = 0.0007; F(2, 100) = 14.86, P = 0.0001]. At baseline (pre-treatment), TDP-43-vehicle and TDP-43-senolytic mice had similar cranial MEP amplitudes (0.002 ± 0.006 and 0.004 ± 0.006 mV/CMAP, P = 0.8843), both lower than controls (0.02 ± 0.01 mV/CMAP, P < 0.0001). It is important to note, that a majority of the TDP-43 mice had MEP amplitudes that were undetectable or very close to 0. By the final time point (six treatment cycles), TDP-43-vehicle remained impaired compared to controls (0.007 ± 0.007 vs. 0.02 ± 0.005 mV/CMAP, P = 0.0107), while TDP-43-senolytic improved (0.02 ± 0.02 mV/CMAP, P = 0.0030) and matched controls (P = 0.8865).

At the final time point (six treatment cycles), we then aimed to corroborate these electrophysiological results with neuronal density in layer V of the PMC using the CTIP2 antibody. CTIP2 staining in PMC layer V (Fig. 4B and E) showed a significant group effect [F(2,9) = 7.562, P = 0.0118]. TDP-43-vehicle mice had lower neuronal density (242.7 ± 69.55) than controls (318.6 ± 71.22, P = 0.0182). TDP-43-senolytic mice had higher density (315.1 ± 63.18) than TDP-43-vehicle (P = 0.0233), matching controls (P = 0.9860).

TDP-43 staining (Fig. 4C and E) showed a significant group effect [F(2, 9) = 50.34, P < 0.0001]. At the final time point (six treatment cycles), TDP-43-vehicle mice had higher MFI (15.32 ± 3.807) than controls (3.631 ± 2.078, P < 0.0001). TDP-43-senolytic mice had lower MFI (9.944 ± 4.227) than TDP-43-vehicle (P = 0.0033) but remained elevated vs. controls (P = 0.0011).

TDP-43/CTIP2 colocalization in layer V (Fig. 4D and E) showed a significant group effect [F(2,9) = 8.419, P = 0.0087]. At the final time point (six treatment cycles), TDP-43-vehicle mice had higher colocalization (0.01488 ± 0.009031) than controls (0.004238 ± 0.003316, P = 0.0085). TDP-43-senolytic mice had lower colocalization (0.007000 ± 0.002996) than TDP-43-vehicle (P = 0.0393) and matched controls (P = 0.5860). Immunofluorescence (Fig. 4E) qualitatively showed reduced CTIP2-positive neurons and increased TDP-43 signal in TDP-43-vehicle mice, while TDP-43-senolytic mice had higher CTIP2-positive neurons and a lower TDP-43 signal, and neuronal numbers similar to control levels.

### 3.3 The senolytics D&Q reduce senescence in the cortex of TDP-43^Q331K^ mice.

**Figure 5:**
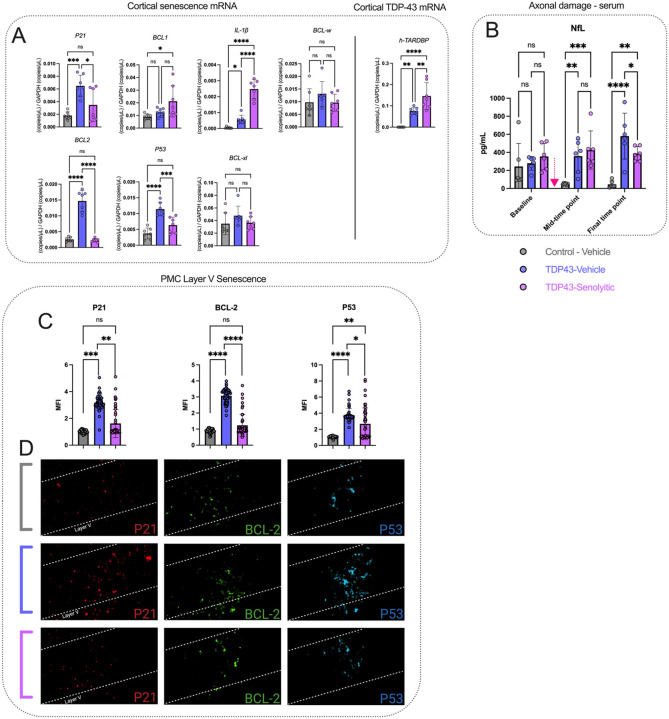
Senolytic treatment reduces cortical senescence and TDP-43 expression, while stabilizing systemic axonal damage in TDP-43^Q331K^ mice. **(A) Cortical senescence** markers (*P21, BCL1, IL-lβ, BCL-w, BCL2, P53,* and *Bcl-xL*) were quantified using digital PCR and normalized to *GAPDH*. TDP-43-vehicle mice exhibited elevated levels of *P21, BCL2, P53,* and *IL1β* compared to controls. Treatment with senolytics significantly reduced P21, *BCL2,* and *P53* levels compared to TDP-43-vehicle mice normalizing these markers to control levels, while *IL-lβ* remained elevated. *BCL1* was elevated in TDP-43-senolytic mice compared to controls but showed no significant difference from TDP-43-vehicle mice. Cortical TDP-43 expression, assessed via human TDP-43 *(h-TARDBP)* transcript levels, was significantly elevated in TDP-43-vehicle and TDP-43-senolytic mice compared to controls, with TDP-43-senolytic mice showing further increases. (B) Axonal damage, represented by serum neurofilament light (NfL) levels, was significantly elevated in TDP-43-vehicle mice compared to controls at the mid-time point and at the final time point. Senolytic treatment significantly reduced NfL levels at the final time point compared to TDP-43-vehicle mice, though levels remained higher than controls. (C-D) P21 (red), a marker of cell-cycle arrest, is significantly increased in TDP-43-vehicle mice compared to controls. Senolytic treatment significantly reduces P21 expression, and normalizes levels compared to controls. BCL2 (green), an anti-apoptotic protein associated with senescence, is significantly increased in TDP-43-vehicle mice compared to controls. Senolytic treatment significantly reduces BCL2 expression, and normalizes levels compared to controls. P53 (cyan), a tumor suppressor and regulator of cellular senescence, is significantly elevated in TDP-43-vehicle mice compared to controls. Senolytic treatment significantly reduces P53 expression, but levels remain elevated compared to controls. Representative images (bottom) show P21, BCL2, and P53 staining in layer V. Significance: *P < 0.05, **P < 0.01, ****P < 0.0001, ns = not significant. Data are presented as means ± SD. A pink arrow indicates when mice received either vehicle or D&Q. If no pink arrow is present, measurements were done terminally at the final time point (Panels A, C, and D).

We used dPCR analysis to measure cortical senescence transcripts in the cortex (Fig. 5A) at the final time point (six treatment cycles). *P21,* involved in cell-cycle arrest, showed a group effect [F(2,18) = 11.86, P = 0.0005], with TDP-43-vehicle mice having elevated *P21* (0.006487 ± 0.001682) vs. controls (0.001808 ± 0.000575, P = 0.0004). Senolytics reduced *P21* (0.003478 ± 0.002605, P = 0.0165), restoring it to control levels (P = 0.2270).

*BCL1,* a cell-cycle regulator, showed a group effect [F(2,18) = 4.901, P = 0.0200]. TDP-43-vehicle levels (0.01248 ± 0.00319) were similar to controls (0.00900 ± 0.00210, P = 0.1030), but senolytics increased *BCL1* (0.02124 ± 0.01247, P = 0.0185).

*IL-1/β*, a neuroinflammation marker, showed a group effect [F(2,18) = 80.38, P< 0.0001]. TDP-43-vehicle mice had elevated *IL-1β* (0.0005697 ± 0.00002668) vs. controls (0.00004532 ± 0.000004961, P = 0.0004). Senolytics further increased *IL-lβ* (0.002474 ± 0.0005943) vs. TDP-43-vehicle (P < 0.0001) and controls (P = 0.0454).

*BCL2,* another anti-apoptotic protein, showed a group effect [F(2,18) = 87.44, P < 0.0001]. TDP-43-vehicle mice had elevated BCL2 (0.01469 ± 0.003360) vs. controls (0.002547 ± 0.000862, P < 0.0001). Senolytics reduced *BCL2* (0.002293 ± 0.000504, P < 0.0001), restoring it to control levels (P = 0.9696).

*P53,* a tumor suppressor, showed a group effect [F(2,18) = 23.45, P < 0.0001]. TDP-43-vehicle mice had higher P53 (0.01138 ± 0.002066) vs. controls (0.003714 ± 0.001734, P < 0.0001). Senolytics reduced P53 (0.006336 ± 0.002517, P = 0.0009), approaching control levels (P = 0.0810).

*BCL-xl* and *BCL-w,* apoptosis regulators, showed no significant group effects [F(2,18) = 1.617, P = 0.2260; F(2,18) = 1.276, P = 0.3033]. No differences were observed across groups.

*hTARDBP,* linked to ALS, showed a group effect [F(2,18) = 26.42, P < 0.0001]. TDP-43-vehicle mice had elevated *hTARDBP* (0.07599 ± 0.01599) vs. controls (5.647 × 10^−6^ ± 7.976 × 10^−6^, P = 0.0037). Senolytics further increased *hTARDBP*(0.1459 ± 0.06305) vs. TDP-43-vehicle (P = 0.0071) and controls (P < 0.0001).

NfL, an axonal damage marker, was measured in serum (Fig. 5B) across 3 time points. A two-way ANOVA showed a significant interaction between factors (group and time) [F(4,45) = 3.987, P = 0.0075] and group main effect [F(2,45) = 18.16, P< 0.0001], though time alone was not a significant main effect [F(2,45) = 0.6318, P = 0.5363]. At the mid-time point (three treatment cycles), TDP-43-vehicle mice had higher NfL (358.9 ± 179.2) vs. controls (50.14±8.3, P = 0.0042). TDP-43-senolytic mice also had elevated NfL (427.0 ±211.4, P = 0.0007), with no difference vs. TDP-43-vehicle (P = 0.4758). At the final time point (six treatment cycles), NfL was higher in TDP-43-vehicle (580.8 ± 254.2) vs. controls (45.56 ± 39.20, P < 0.0001), while senolytics reduced NfL (386.0 ± 79.1) vs. TDP-43-vehicle (P = 0.0454) but remained higher than controls (P = 0.0016).

We then used immunohistochemistry to assess protein expression in PMC layer V and confirm transcriptional findings (Fig. 5C) at the final time point (six treatment cycles). P21 MFI showed a group effect [F(2,9) = 21.61, P = 0.0004], with TDP-43-vehicle mice having higher P21 (3.173 ± 0.5580) vs. controls (1.023 ±0.1178, P = 0.0004). Senolytics reduced P21 (1.621 ± 1.051, P = 0.0028), normalizing it (P = 0.2925). BCL2 MFI showed a group effect [F(2,9) = 80.93, P < 0.0001], with TDP-43-vehicle mice showing increased BCL2 (3.173±0.4416) vs. controls (1.023±0.1275, P< 0.0001). Senolytics lowered BCL2 (1.569 ± 0.6904, P < 0.0001), normalizing it (P = 0.2630). P53 MFI showed a group effect [F(2,9) = 39.09, P < 0.0001], with TDP-43-vehicle mice having higher P53 (3.776 ± 0.8125) vs. controls (1.050 ± 0.1011, P< 0.0001). Senolytics reduced P53 (2.657 ± 1.977, P = 0.0138) but remained higher than controls (P = 0.0015).

Qualitatively, immunofluorescence (Fig. 5D) showed increased P21, BCL2, and P53 in TDP-43-vehicle mice. Senolytic-treated mice had lower levels, indicating reduced senescence and pathology.

### 3.4 The senolytics D&Q reduce microglial proliferation, senescence, and ALS pathology in TDP-43^Q331K^ mice.

**Figure 6:**
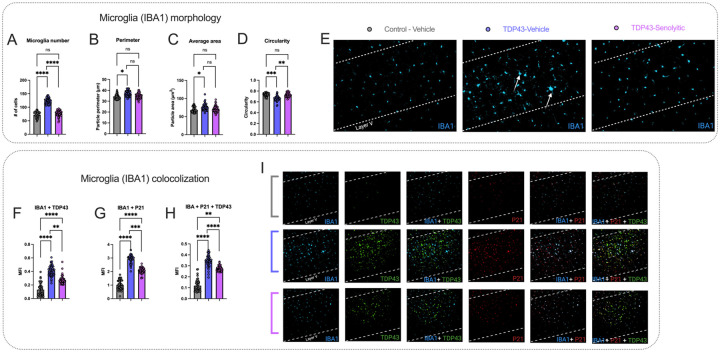
Senolytic treatment reduces microglial activation, senescence, and TDP-43 colocalization in TDP-43^Q331K^ mice. **(A-E).** TDP-43-vehicle mice showed increased microglial density **(A)**, larger cell perimeter **(B)**, and increased cell area **(C)** compared to controls. Senolytics reduced microglial density and restored morphology parameters to control levels. Circularity **(D)** was reduced in TDP-43-vehicle mice compared to controls and improved with senolytics, normalizing to control levels. **(E)** Representative immunofluorescence images of IBA1 staining in layer V show increased microglial numbers and altered morphology in TDP-43-vehicle mice (white arrows), compared to controls. These changes are reduced in TDP-43-senolytic mice. **(F-I)**. IBA1+TDP43 **(F)** IBA1+P21 **(G)** and IBA1+P21+TDP43 **(H)** colocalization showed significant increases in TDP-43-vehicle mice compared to controls. Senolytic treatment reduced colocalization across all markers, though all values remained higher than controls. **(I)** Representative images of IBA1+TDP43, IBA1+P21, and IBA1+P21+TDP43 colocalization show increased signals in TDP-43-vehicle mice, which decrease with senolytic treatment. Significance: *P < 0.05, **P < 0.01, ****P < 0.0001, ns = not significant. Data are presented as means ± SD. All measurements were done terminally at the final time point.

Since microglia have been implicated in ALS spread and senescent microglia can be effectively targeted by senolytics [21, 35], we also assessed microglial morphology [33, 36], senescence, and TDP-43 pathology in layer V of the PMC (Fig. 6) at the final time point (six treatment cycles). IBA1-positive cells showed a group effect [F(2,9) = 73.93, P < 0.0001], with TDP-43-vehicle mice having higher microglial density (123.2 ± 10.49) than controls (72.56 ± 10.80, P < 0.0001). Senolytics reduced density (77.07 ± 10.47, P < 0.0001), matching control levels (P = 0.6127). Average area of IBA1-positive cells also showed a significant group effect [F(2,9) = 4.161, P = 0.0425]. TDP-43-vehicle mice had larger areas (76.34 μm^2^ ± 9.435) than controls (68.66 μm^2^ ± 6.900, P = 0.0446), with no difference between TDP-43-vehicle and senolytic-treated mice (71.70 μm^2^ ±8.395, P = 0.5235). Average perimeter of IBA1-positive cells showed a significant group effect [F(2,9) = 6.680, P = 0.0166], with TDP-43-vehicle mice having larger perimeters (37.11 μm± 2.550) than controls (34.11 μm± 2.152, P = 0.0149). No difference was observed between senolytic-treated and TDP-43-vehicle mice (P = 0.5378).

Circularity of IBA1-positive cells showed a significant group effect [F(2,9) = 19.70, P = 0.0005]. TDP-43-vehicle mice had lower circularity (0.6628 ± 0.04721) compared to controls (0.7347 ± 0.02980, P = 0.0008). Senolytics increased circularity (0.7276 ± 0.0363, P = 0.0016), matching control levels (P = 0.8436). Qualitatively, immunofluorescence (Fig. 6E) shows increased IBA1-positive cells with larger morphologies in TDP-43-vehicle mice compared to controls (white arrows). These changes are reduced in TDP-43-senolytic mice.

We then quantified IBA1 + P21 colocalization MFI, which showed a significant group effect [F(2,9) = 122.0, P < 0.0001]. TDP-43-vehicle mice had higher MFI (2.922 ± 0.3020) than controls (1.021 ± 0.3483, P < 0.0001). Senolytics reduced MFI (2.072 ± 0.2192, P = 0.0002) but levels remained higher than controls (P < 0.0001). IBA1 + TDP43 colocalization MFI also showed a significant group effect [F(2,9) = 42.45, P < 0.0001], with TDP-43-vehicle mice having higher levels (0.4126 ± 0.08238) than controls (0.1307 ± 0.08650, P < 0.0001). Senolytics reduced MFI (0.2752 ± 0.06010, P = 0.0002), though it remained higher than controls (P < 0.0001). Finally, IBA1 + P21 + TDP43 colocalization MFI showed a significant group effect [F(2,9) = 90.56, P < 0.0001]. TDP-43-vehicle mice had higher MFI (0.3452 ± 0.05260) than controls (0.1189 ± 0.05264, P < 0.0001). Senolytics reduced MFI (0.2751 ± 0.02330, P < 0.0001) but levels remained higher than controls (P = 0.0017). Qualitatively, immunofluorescence (Fig. 6I) shows increased IBA1 + TDP43, IBA1 + P21, and IBA1 + P21 + TDP43 colocalization in TDP-43-vehicle mice compared to controls. Colocalization decreases in TDP-43-senolytic mice.

## 4. DISCUSSION

This study is the first to investigate the impact of senolytic treatment in an ALS model. We show that over the course of fifteen weeks (Fig. 1A), D&Q treatment improved neuromuscular function (Fig. 2–3) and stabilized neuronal damage (Fig. 5B) in TDP-43^Q331K^ mice. Notably, the most pronounced improvement was the preservation of cortical excitability, approximating control levels of excitability (Fig. 4A), which was accompanied by concomitant improvements in layer V of the PMC, including preserved neuronal number, reduced senescence, and decreased TDP-43 accumulation (Fig. 4). Additionally, D&Q reduced the proliferation, senescence, and altered morphology of microglia, indicating decreased microglial activation and suggesting beneficial effects on both neurons and their supporting glial cells (Fig. 6). Our findings suggest that targeting senescence, mitigates both neuromuscular and cortical dysfunction.

These results align with growing evidence that biological aging plays a critical role in ALS neurodegeneration [2, 5, 37, 38]. Biological aging, the progressive decline in cellular function over time, is a normal aspect of chronological aging and involves molecular alterations such as DNA damage, epigenetic modifications, mitochondrial dysfunction, and chronic inflammation [2–4]. In healthy individuals, biological and chronological aging progress in parallel. However, when biological aging outpaces chronological aging, it can contribute to neurodegenerative pathologies, including Alzheimer’s disease [38], Parkinson’s Disease [39], traumatic-brain injury [21], and Huntington’s disease [40].

In ALS, accelerated biological aging[2, 7, 8] can trigger cellular senescence [41, 42], which disrupts the neural microenvironment, drives chronic inflammation, and propagates dysfunction to neighboring cells [2, 7]. For example, astrocytes expressing the senescence markers P16 and P21[6] have been detected in post-mortem ALS patient tissues, while microglia-like cells derived from sporadic ALS patients exhibit a senescence-associated neuroinflammatory phenotype [43]. Similarly, senescent lymphocytes have been identified in ALS patients [13], and SOD1 mouse models display increased senescence markers during paralysis progression [41]. These lines of evidence in ALS suggest that senescence may be linked to disease progression [7].

Building on this link between senescence and ALS, senolytic drugs, which selectively clear senescent cells, may offer a promising intervention. D&Q, a well-studied senolytic combination, has been shown to eliminate senescent cells across multiple disease models [14–16, 20]. In Alzheimer’s disease [20], senolytics reduced senescent glial cells and improved synaptic function and memory, as well as in traumatic brain injury [21], osteoporosis [44], cardiovascular disease [45], and others. Given this broad therapeutic potential and the strong link between aging and ALS pathology, we tested the effects of D&Q in TDP-43^Q331K^ mice.

Our findings show that D&Q improved motor strength and coordination in TDP-43^Q331K^ mice (Fig. 2), associated with neuromuscular (lower motor neuron / peripheral nervous system) functional improvement (Fig. 3), such as increased muscular excitability (CMAPs), partial rescue of motor unit number (MUNE), and increased plantar force production (twitch and tetanic). These improvements coincided with stabilization of NfL serum levels throughout the course of D&Q treatment, suggesting preservation of axonal integrity (Fig. 5B). NfL is a widely recognized clinical biomarker of disease progression in ALS [46]. Notably, reductions in NfL levels have been associated with slowed disease progression in ALS patients receiving the antisense oligonucleotide therapy Tofersen for SOD1-driven ALS [47, 48], making our findings particularly relevant in the context of senolytic treatment.

To evaluate the impact of D&Q beyond neuromuscular function, we also examined cortical excitability, a critical component of motor dysfunction and pathogenesis in ALS [1, 49, 50]. Increasing evidence shows that ALS pathology may originate in the PMC and propagate along the nervous system [49, 51–53]. In both SOD1 animal models and ALS patients, early cortical excitability changes have been shown to precede spinal motor neuron and neuromuscular degeneration [54, 55], with similar trends observed in TDP-43 models [51, 56], reinforcing the role of cortical dysfunction in ALS progression. Our findings show a profound reduction in cortical excitability (Fig. 4A)—measured through lower limb gastrocnemius muscle recordings—to near-undetectable levels early in disease progression, making it the most severe electrophysiological deficit in this model.

Despite the significant cortical dysfunction observed in our study and others, few therapeutics are able to directly target these abnormalities. Riluzole, for example, modulates neurotoxic changes in cortical excitability [57], though its effects are modest and transient [58]. Similarly, ezogabine has shown some improvements in ALS-related excitability changes, but with limited clinical impact [58]. Remarkably, D&Q treatment preserved cortical excitability at control levels (Fig. 4), representing the most significant improvement in our study. This was accompanied also by the maintenance of neuronal counts at control levels (Fig. 4B), suggesting that D&Q may enhance neuronal survivability and thereby supporting cortical function. These findings highlight the neuroprotective role of D&Q in cortical health and excitability.

Furthermore, while our findings align with ALS patient studies reporting hypoexcitability in lower limb motor areas [59], they also contrast with previous evidence in both humans and mice, which frequently reports cortical hyperexcitability in ALS. For instance, TDP-43 [51] and SOD1 [52] mouse models typically exhibit hyperexcitability due to excitotoxicity-driven neuronal dysfunction [50]. However, these conclusions are largely based on in-vitro patch-clamp recordings of layer V pyramidal neurons. Similarly, transcranial magnetic stimulation (TMS) studies in ALS patients often report cortical hyperexcitability [60] yet many also reveal limb-dependent differences, with hyperexcitability in upper limb motor areas [58] and hypoexcitability in lower limb regions [59]. This variability suggests that ALS induces complex, region-specific changes in cortical excitability that manifest differently depending on the method of measurement.

Our findings also show that cortical improvement is linked to reduction in cortical senescence (Fig. 5). Transcriptional analyses revealed increased *P21, IL1B, BCL2, and P53* in the cortex of vehicle-treated mice, indicative of senescence-induced cell-cycle arrest, impaired apoptosis, and neuroinflammation [6, 9, 10, 61, 62]. D&Q treatment restored *P21, BCL2, and P53* expression to control levels, suggesting a reduced senescence burden. Immunohistochemical analyses confirmed transcript-level findings, with increased P21, BCL2, and P53 protein expression in PMC layer V of vehicle-treated mice, and D&Q normalizing P21 and BCL2 while reducing P53. Although few studies have examined the impact of senescence markers on neuronal excitability [63, 64], our findings emphasize the need for further investigation on how specific senescence markers affect neuronal function and their potential as therapeutic targets in ALS.

While our results highlight the potential of senolytics in mitigating cortical senescence, they also raise key questions about their impact on TDP-43 pathology. In vehicle-treated mice, TDP-43 levels were strongly colocalized with layer V neurons, whereas D&Q treatment reduced TDP-43 protein levels (Fig. 4C–E). Interestingly, despite this reduction, transcriptional data showed increased cortical TDP-43 transcripts, suggesting a compensatory response to protein clearance. This raises the possibility that the enhanced neuronal survivability observed with D&Q may be linked to the clearance of TDP-43 from the cortex. Prior studies show that activated microglia can clear TDP-43 from the spinal cord and improve motor function, while astrocytes co-cultured with motor neurons can internalize cytoplasmic TDP-43 [65–67]. To build on this and explore potential mechanisms underlying our results, we quantified microglia in PMC layer V (Fig. 6). TDP-43 vehicle-treated mice exhibited increased microglial proliferation and morphological changes, including decreased circularity, and increased area and perimeter—indicative of activation in response to immune challenges and ALS pathology [33, 36, 43, 66, 68]. These microglia also showed increased colocalization with P21 [21] and TDP-43, confirming a high senescent microglial burden in PMC layer V, which may contribute to the reduced cortical neuronal numbers we observed [66]. In contrast, senolytic treatment reduced microglial proliferation to control levels as well as reducing morphological changes. This was also accompanied by decreased colocalization with P21, indicating that D&Q reduces senescence in microglia. These findings demonstrate that senolytics alleviate senescence in cells other than neurons and may induce the apoptosis of senescent microglia as reported in other studies [21].

Given this, cortical improvements may not be directly driven by microglial clearance of TDP-43 but instead result from indirect effects. We propose that microglia may facilitate the spread of TDP-43 pathology throughout the brain [67, 69], and senolytics may aid in blunting this process. It is plausible that senolytics may be clearing senescent microglia, as indicated by the reduction in microglia colocalized with P21. This reduction in senescent microglia may further suppress activation of surrounding microglia and limit their role in propagating TDP-43 dysfunction, as reflected in the decreased TDP-43 signal in layer V following senolytic treatment. However, this does not exclude the possibility that microglia or astrocytes play a direct role in the functional improvements observed in ALS after senolytic therapy. Future studies will be necessary to determine whether senolytics mitigate TDP-43 pathology by reducing the ability of glial cells to spread TDP-43.

In summary, here we demonstrate the neuroprotective potential of senolytics in ALS by improving neuromuscular function, preserving cortical excitability, and reducing senescence. These findings highlight the role of aging in ALS pathology and support further investigation into senolytics as a therapeutic strategy.

## Data Availability

Data will be made available upon reasonable request.

## References

[1] Masrori P, Van Damme P (2020) Amyotrophic lateral sclerosis: a clinical review. Eur J Neurol 27:1918–1929 32526057 10.1111/ene.14393 PMC7540334

[2] Dashtmian AR, Darvishi FR, Arnold WD (2024) Chronological and Biological Aging in Amyotrophic Lateral Sclerosis and the Potential of Senolytic Therapies. Cells ;13 10.3390/cells13110928 PMC11171952 38891059

[3] Das MM, Svendsen CN (2015) Astrocytes show reduced support of motor neurons with aging that is accelerated in a rodent model of ALS. Neurobiol Aging 36:1130–1139 25443290 10.1016/j.neurobiolaging.2014.09.020

[4] Cuollo L, Antonangeli F, Santoni A, Soriani A (2020) The Senescence-Associated Secretory Phenotype and Age-Related Diseases. Biology (Basel) 9:1–16 10.3390/biology9120485 PMC7767554 33371508

[5] Hamczyk MR, Nevado RM, Barettino A, Fuster V, Andrés V (2020) Biological Versus Chronological Aging: JACC Focus Seminar. J Am Coll Cardiol 75:919–930 32130928 10.1016/j.jacc.2019.11.062

[6] Vazquez-Villaseñor I, Garwood CJ, Heath PR, Simpson JE, Ince PG, Wharton SB (2020) Expression of p16 and p21 in the frontal association cortex of ALS/MND brains suggests neuronal cell cycle dysregulation and astrocyte senescence in early stages of the disease. Neuropathol Appl Neurobiol 46:171–185 31077599 10.1111/nan.12559 PMC7217199

[7] Maximova A, Werry EL, Kassiou M, (2021) Senolytics A novel strategy for neuroprotection in als? Int J Mol Sci 22:1–15 10.3390/ijms222112078 PMC8584291 34769512

[8] Pandya VA, Patani R (2020) Decoding the relationship between ageing and amyotrophic lateral sclerosis: A cellular perspective. Brain 143:1057–1072. 10.1093/brain/awz360 31851317 PMC7174045

[9] Baar MP, Brandt RMC, Putavet DA, Klein JDD, Derks KWJ, Bourgeois BRM (2017) Targeted Apoptosis of Senescent Cells Restores Tissue Homeostasis in Response to Chemotoxicity and Aging. Cell 169:132–147 28340339 10.1016/j.cell.2017.02.031 PMC5556182

[10] Matsudaira T, Nakano S, Konishi Y, Kawamoto S, Uemura K, Kondo T (2023) Cellular senescence in white matter microglia is induced during ageing in mice and exacerbates the neuroinflammatory phenotype. Commun Biol 6:2–8 37353538 10.1038/s42003-023-05027-2 PMC10290132

[11] Sikora E, Bielak-Zmijewska A, Dudkowska A, Krzystyniak A, Mosieniak G, Wesierska M (2021) Cell Senescence Brain Aging Front Aging Neurosci 13:1–23 10.3389/fnagi.2021.646924 PMC7959760 33732142

[12] Tan X, Su X, Wang Y, Liang W, Wang D, Huo D (2024) BRD7 regulates cellular senescence and apoptosis in ALS by modulating p21 expression and p53 mitochondrial translocation respectively. Neuroscience 563:51–62. 10.1016/j.neuroscience.2024.11.004 39510439

[13] Yildiz O, Schroth J, Tree T, Turner MR, Shaw PJ, Henson SM (2023) Senescent-like Blood Lymphocytes and Disease Progression in Amyotrophic Lateral Sclerosis. Neurol Neuroimmunol Neuroinflamm 10:1–13. 10.1212/NXI.0000000000200042 PMC9673751 36323511

[14] Xu M, Pirtskhalava T, Farr JN, Weigand BM, Palmer AK, Weivoda MM (2018) Senolytics improve physical function and increase lifespan in old age. Nat Med 24:1246–1256 29988130 10.1038/s41591-018-0092-9 PMC6082705

[15] Aguado J, Amarilla AA, Taherian Fard A, Albornoz EA, Tyshkovskiy A, Schwabenland M (2023) Senolytic therapy alleviates physiological human brain aging and COVID-19 neuropathology. Nat Aging10.1038/s43587-023-00519-6 PMC10724067 37957361

[16] Islam M, Tuday ER, Allen S, Kim J, Trott DW, Holland WL (2023) Senolytic drugs, dasatinib and quercetin, attenuate adipose tissue inflammation, and ameliorate metabolic function in old age. Aging Cell 22:1–15 10.1111/acel.13767 PMC9924942 36637079

[17] Yi Zhu, Tchkonia T, Pirtskhalava T, Gower AC, Ding H, Giorgadze N (2015) The achilles’ heel of senescent cells: From transcriptome to senolytic drugs. Aging Cell 14:644–658. 10.1111/acel.12344 25754370 PMC4531078

[18] Kirkland JL, Tchkonia T (2020) Senolytic drugs: from discovery to translation. J Intern Med 288:518–536. 10.1111/joim.13141 32686219 PMC7405395

[19] Rad AN, Grillari J (2024) Current senolytics: Mode of action, efficacy and limitations, and their future. Mech Ageing Dev 217:111888. 10.1016/j.mad.2023.111888 38040344

[20] Zhang P, Kishimoto Y, Grammatikakis I, Gottimukkala K, Cutler RG, Zhang S (2019) Senolytic therapy alleviates Aβ-associated oligodendrocyte progenitor cell senescence and cognitive deficits in an Alzheimer’s disease model. Nat Neurosci 22:719–728 30936558 10.1038/s41593-019-0372-9 PMC6605052

[21] Wang J, Lu Y, Carr C, Dhandapani KM, Brann DW (2023) Senolytic therapy is neuroprotective and improves functional outcome long-term after traumatic brain injury in mice. Front Neurosci 17:1–15. 10.3389/fnins.2023.1227705 PMC10416099 37575310

[22] Ogrodnik M, Zhu Y, Langhi LGP, Tchkonia T, Krüger P, Fielder E (2019) Obesity-Induced Cellular Senescence Drives Anxiety and Impairs Neurogenesis. Cell Metab 29:1061–1077e8. 10.1016/j.cmet.2018.12.008 30612898 PMC6509403

[23] Watkins JA, Alix JJP, Shaw PJ, Mead RJ (2021) Extensive phenotypic characterisation of a human TDP-43Q331K transgenic mouse model of amyotrophic lateral sclerosis (ALS). Sci Rep 11:1–14. 10.1038/s41598-021-96122-z 34404845 PMC8370970

[24] Mitchell JC, Constable R, So E, Vance C, Scotter E, Glover L (2015) Wild type human TDP-43 potentiates ALS-linked mutant TDP-43 driven progressive motor and cortical neuron degeneration with pathological features of ALS. Acta Neuropathol Commun 3:36. 10.1186/s40478-015-0212-4 26108367 PMC4479086

[25] Owendoff G, Ray A, Bobbili P, Clark L, Baumann CW, Clark BC (2023) Optimization and construct validity of approaches to preclinical grip strength testing. J Cachexia Sarcopenia Muscle 14:2439–2445 37574215 10.1002/jcsm.13300 PMC10570062

[26] Kerr NR, Dashtmian AR, Darvishi FB, Brennan CD, Ayyagari SN, Moore PJ 5-HT2C agonism as a neurotherapeutic for sarcopenia: preclinical proof of concept. Geroscience 2025. 10.1007/s11357-025-01519-7 PMC12181463 39825167

[27] Arnold WD, Sheth KA, Wier CG, Kissel JT, Burghes AH, Kolb SJ (2015) Electrophysiological motor unit number estimation (MUNE) measuring compound muscle action potential (CMAP) in mouse hindlimb muscles. J Visualized Experiments :1–8 10.3791/52899 PMC4676269 26436455

[28] Castoldi V, Rossi E, Marenna S, Comi G, Leocani L (2022) Improving reproducibility of motor evoked potentials in mice. J Neurosci Methods 367:109444 34921842 10.1016/j.jneumeth.2021.109444

[29] Steward O, Yee KM, Metcalfe M, Willenberg R, Luo J, Azevedo R (2021) Rostro-Caudal Specificity of Corticospinal Tract Projections in Mice. Cereb Cortex 31:2322–2344 33350438 10.1093/cercor/bhaa338 PMC8023844

[30] Wier CG, Crum AE, Reynolds AB, Iyer CC, Chugh D, Palettas MS (2019) Muscle contractility dysfunction precedes loss of motor unit connectivity in SOD1(G93A) mice. Muscle Nerve 59:254–262. 10.1002/mus.26365 30370671 PMC6340745

[31] Sheth KA, Iyer CC, Wier CG, Crum AE, Bratasz A, Kolb SJ (2018) Muscle strength and size are associated with motor unit connectivity in aged mice. Neurobiol Aging 67:128–136 29656012 10.1016/j.neurobiolaging.2018.03.016 PMC5981861

[32] Kerr NR, Kelty TJ, Mao X, Childs TE, Kline DD, Rector RS (2023) Selective breeding for physical inactivity produces cognitive deficits via altered hippocampal mitochondrial and synaptic function. Front Aging Neurosci 15. 10.3389/fnagi.2023.1147420 PMC10106691 37077501

[33] Green TRF, Murphy SM, Rowe RK (2022) Comparisons of quantitative approaches for assessing microglial morphology reveal inconsistencies, ecological fallacy, and a need for standardization. Sci Rep 12:1–13. 10.1038/s41598-022-23091-2 36307475 PMC9616881

[34] Viteri JA, Bueschke N, Santin JM, Arnold WD (2024) Age-related increase in the excitability of mouse layer V pyramidal neurons in the primary motor cortex is accompanied by an increased persistent inward current. Geroscience. 10.1007/s11357-024-01405-8 PMC11979039 39472350

[35] Drake S, Zaman A, Gianfelice C, Hua EML, Heale K, Afanasiev E Senolytic treatment depletes microglia and decreases severity of experimental autoimmune encephalomyelitis. BioRxiv 2024:2024.02.05.579017. 10.1186/s12974-024-03278-2 PMC11529445 39487537

[36] Davis BM, Salinas-Navarro M, Cordeiro MF, Moons L, Groef LD (2017) Characterizing microglia activation: A spatial statistics approach to maximize information extraction. Sci Rep 7:1–12. 10.1038/s41598-017-01747-8 28484229 PMC5431479

[37] Ferrucci L, Gonzalez-Freire M, Fabbri E, Simonsick E, Tanaka T, Moore Z (2020) Measuring biological aging in humans: A quest. Aging Cell 19:1–21 10.1111/acel.13080 PMC6996955 31833194

[38] Gonzales MM, Garbarino VR, Pollet E, Palavicini JP, Kellogg DL, Kraig E (2022) Biological aging processes underlying cognitive decline and neurodegenerative disease. J Clin Invest 132. 10.1172/JCI158453 PMC9106343 35575089

[39] Chinta SJ, Woods G, Demaria M, Rane A, Zou Y, McQuade A (2018) Cellular Senescence Is Induced by the Environmental Neurotoxin Paraquat and Contributes to Neuropathology Linked to Parkinson’s Disease. Cell Rep 22:930–940. 10.1016/j.celrep.2017.12.092 29386135 PMC5806534

[40] Horvath S, Langfelder P, Kwak S, Aaronson J, Rosinski J, Vogt TF (2016) Huntington’s disease accelerates epigenetic aging of human brain and disrupts DNA methylation levels. Aging 8:1485–1512. 10.18632/aging.101005 27479945 PMC4993344

[41] Trias E, Beilby PR, Kovacs M, Ibarburu S, Varela V, Barreto-Núñez R (2019) Emergence of microglia bearing senescence markers during paralysis progression in a rat model of inherited ALS. Front Aging Neurosci 10:1–14 10.3389/fnagi.2019.00042 PMC6403180 30873018

[42] Jagaraj CJ, Shadfar S, Kashani SA, Saravanabavan S, Farzana F, Atkin JD (2024) Molecular hallmarks of ageing in amyotrophic lateral sclerosis. Cell Mol Life Sci 81. 10.1007/s00018-024-05164-9 PMC10908642 38430277

[43] Quek H, Cuní-López C, Stewart R, Colletti T, Notaro A, Nguyen TH (2022) ALS monocyte-derived microglia-like cells reveal cytoplasmic TDP-43 accumulation, DNA damage, and cell-specific impairment of phagocytosis associated with disease progression. J Neuroinflammation 19:1–21. 10.1186/s12974-022-02421-1 35227277 PMC8887023

[44] Wang S, Zhai J, Heng K, Sha L, Song X, Zhai H (2024) Senolytic cocktail dasatinib and quercetin attenuates chronic high altitude hypoxia associated bone loss in mice. Sci Rep 14:1–13. 10.1038/s41598-024-82262-5 39638948 PMC11621334

[45] Salerno N, Marino F, Scalise M, Salerno L, Molinaro C, Filardo A (2022) Pharmacological clearance of senescent cells improves cardiac remodeling and function after myocardial infarction in female aged mice. Mech Ageing Dev 208:111740. 10.1016/j.mad.2022.111740 36150603

[46] Meyer T, Dreger M, Grehl T, Weyen U, Kettemann D, Weydt P (2024) Serum neurofilament light chain in distinct phenotypes of amyotrophic lateral sclerosis: A longitudinal, multicenter study. Eur J Neurol 31:1–13. 10.1111/ene.16379 PMC11295170 38859579

[47] Meyer T, Schumann P, Weydt P, Petri S, Koc Y, Spittel S (2023) Neurofilament light-chain response during therapy with antisense oligonucleotide tofersen in SOD1-related ALS: Treatment experience in clinical practice. Muscle Nerve 67:515–521. 10.1002/mus.27818 36928619

[48] Wiesenfarth M, Dorst J, Brenner D, Elmas Z, Parlak O, Uzelac Z (2024) Effects of tofersen treatment in patients with SOD1-ALS in a real-world setting – a 12-month multicenter cohort study from the German early access program. EClinicalMedicine 69:102495. 10.1016/j.eclinm.2024.102495 38384337 PMC10878861

[49] Menon P, van den Higashihara M, Geevasinga N, Kiernan MC, Vucic S (2020) Cortical hyperexcitability evolves with disease progression in ALS. Ann Clin Transl Neurol 7:733–741. 10.1002/acn3.51039 32304186 PMC7261748

[50] Xie M, Pallegar PN, Parusel S, Nguyen AT, Wu LJ (2023) Regulation of cortical hyperexcitability in amyotrophic lateral sclerosis: focusing on glial mechanisms. Mol Neurodegener 18:1–21 37858176 10.1186/s13024-023-00665-w PMC10585818

[51] Dyer MS, Reale LA, Lewis KE, Walker AK, Dickson TC, Woodhouse A (2021) Mislocalisation of TDP-43 to the cytoplasm causes cortical hyperexcitability and reduced excitatory neurotransmission in the motor cortex. J Neurochem 157:1300–1315 33064315 10.1111/jnc.15214

[52] Saba L, Viscomi MT, Caioli S, Pignataro A, Bisicchia E, Pieri M (2016) Altered Functionality, Morphology, and Vesicular Glutamate Transporter Expression of Cortical Motor Neurons from a Presymptomatic Mouse Model of Amyotrophic Lateral Sclerosis. Cereb Cortex ;26 10.1093/cercor/bhu317 25596588

[53] Wainger BJ, Kiskinis E, Mellin C, Wiskow O, Han SSW, Sandoe J (2014) Intrinsic membrane hyperexcitability of amyotrophic lateral sclerosis patient-derived motor neurons. Cell Rep 7:1–11 24703839 10.1016/j.celrep.2014.03.019 PMC4023477

[54] Menon P, Kiernan MC (2015) Cortical hyperexcitability precedes lower motor neuron dysfunction in ALS. Clin Neurophysiol 126:803–809 25227219 10.1016/j.clinph.2014.04.023

[55] Marques C, Burg T, Scekic-Zahirovic J, Fischer M, Rouaux C (2021) Upper and lower motor neuron degenerations are somatotopically related and temporally ordered in the SOD1 mouse model of amyotrophic lateral sclerosis. Brain Sci 11:1–18 10.3390/brainsci11030369 PMC7998935 33805792

[56] Reale LA, Dyer MS, Perry SE, Young KM, Dickson TC, Woodhouse A (2023) Pathologically mislocalised TDP-43 in upper motor neurons causes a die-forward spread of ALS-like pathogenic changes throughout the mouse corticomotor system. Prog Neurobiol 226:102449. 10.1016/j.pneurobio.2023.102449 37011806

[57] Vucic S, Lin SHY, Cheah BC, Murray J, Menon P, Krishnan AV (2013) Riluzole exerts central and peripheral modulating effects in amyotrophic lateral sclerosis. Brain 136:1361–1370. 10.1093/brain/awt085 23616585

[58] Wainger BJ, Macklin EA, Vucic S, McIlduff CE, Paganoni S, Maragakis NJ (2021) Effect of Ezogabine on Cortical and Spinal Motor Neuron Excitability in Amyotrophic Lateral Sclerosis: A Randomized Clinical Trial. JAMA Neurol 78:186–196. 10.1001/jamaneurol.2020.4300 33226425 PMC7684515

[59] Menon P, Yiannikas C, Kiernan MC, Vucic S (2019) Regional motor cortex dysfunction in amyotrophic lateral sclerosis. Ann Clin Transl Neurol 6:1373–1382. 10.1002/acn3.50819 31402622 PMC6689694

[60] Vucic S, Ziemann U, Eisen A, Hallett M, Kiernan MC (2013) Transcranial magnetic stimulation and amyotrophic lateral sclerosis: Pathophysiological insights. J Neurol Neurosurg Psychiatry 84:1161–1170. 10.1136/jnnp-2012-304019 23264687 PMC3786661

[61] Wagner KD, Wagner N The Senescence Markers p16INK4A, p14ARF/p19ARF, and p21 in Organ Development and Homeostasis. Cells 2022;11. 10.3390/cells11121966 PMC9221567 35741095

[62] Ho JN, Byun SS, Kim D, Ryu H, Lee S (2024) Dasatinib induces apoptosis and autophagy by suppressing the PI3K/Akt/mTOR pathway in bladder cancer cells. Investig Clin Urol 65:593–602. 10.4111/icu.20240250 PMC11543652 39505519

[63] Wang DX, Dong ZJ, Deng XD, Tian YM, Xiao YJ, Li X GDF11 slows excitatory neuronal senescence and brain ageing by repressing p21. Nat Commun 2023;14. 10.1038/s41467-023-43292-1 PMC10656444 37978295

[64] Bories CY, Arsenault D, Lemire M, Tremblay C, De Koninck Y, Calon F (2017) Transgenic autoinhibition of p21-activated kinase exacerbates synaptic impairments and fronto-dependent behavioral deficits in an animal model of Alzheimer’s disease. Aging 9:1386–1403. 10.18632/aging.101239 28522792 PMC5472739

[65] Smethurst P, Risse E, Tyzack GE, Mitchell JS, Taha DM, Chen YR (2020) Distinct responses of neurons and astrocytes to TDP-43 proteinopathy in amyotrophic lateral sclerosis. Brain 143:430–440. 10.1093/brain/awz419 32040555 PMC7009461

[66] Spiller KJ, Restrepo CR, Khan T, Dominique MA, Fang TC, Canter RG (2018) Microglia-mediated recovery from ALS-relevant motor neuron degeneration in a mouse model of TDP-43 proteinopathy. Nat Neurosci 21:329–340. 10.1038/s41593-018-0083-7 29463850 PMC5857237

[67] Svahn AJ, Don EK, Badrock AP, Cole NJ, Graeber MB, Yerbury JJ (2018) Nucleo-cytoplasmic transport of TDP-43 studied in real time: impaired microglia function leads to axonal spreading of TDP-43 in degenerating motor neurons. Acta Neuropathol 136:445–459. 10.1007/s00401-018-1875-2 29943193 PMC6096729

[68] Migliarini S, Scaricamazza S, Valle C, Ferri A, Pasqualetti M, Ferraro E (2021) Microglia morphological changes in the motor cortex of hsod1g93a transgenic als mice. Brain Sci 11. 10.3390/brainsci11060807 PMC8234003 34207086

[69] Jo M, Lee S, Jeon YM, Kim S, Kwon Y, Kim HJ (2020) The role of TDP-43 propagation in neurodegenerative diseases: integrating insights from clinical and experimental studies. Exp Mol Med 52:1652–1662 33051572 10.1038/s12276-020-00513-7 PMC8080625

